# Quantitative MRI evaluation of articular cartilage in patients with meniscus tear

**DOI:** 10.3389/fendo.2022.911893

**Published:** 2022-07-29

**Authors:** Enqi Chen, Wenjing Hou, Hu Wang, Jing Li, Yangjing Lin, He Liu, Mingshan Du, Lian Li, Xianqi Wang, Jing Yang, Rui Yang, Changru Zhou, Pinzhen Chen, Meng Zeng, Qiandong Yao, Wei Chen

**Affiliations:** ^1^ Department of Radiology, Southwest Hospital, Army Medical University (Third Military Medical University), Chongqing, China; ^2^ Department of Radiology, Sichuan Science City Hospital, Mianyang, China; ^3^ Centre of Joint Surgery, Southwest Hospital, Army Medical University (Third Military Medical University), Chongqing, China

**Keywords:** articular cartilage, meniscus tear, quantitative, T2 mapping, cartilage thickness, MRI, cartilage volume

## Abstract

**Purpose:**

The aim of this study was to assess quantitatively articular cartilage volume, thickness, and T2 value alterations in meniscus tear patients.

**Materials and methods:**

The study included 32 patients with meniscus tears (17 females, 15 males; mean age: 40.16 ± 11.85 years) and 24 healthy controls (12 females; 12 males; mean age: 36 ± 9.14 years). All subjects were examined by 3 T magnetic resonance imaging (MRI) with 3D dual-echo steady-state (DESS) and T2 mapping images. All patients underwent diagnostic arthroscopy and treatment. Cartilage thickness, cartilage volume and T2 values of 21 subregions of knee cartilage were measured using the prototype KneeCaP software (version 2.1; Siemens Healthcare, Erlangen, Germany). Mann-Whitney-U tests were utilized to determine if there were any significant differences among subregional articular cartilage volume, thickness and T2 value between patients with meniscus tear and the control group.

**Results:**

The articular cartilage T2 values in all subregions of the femur and tibia in the meniscus tear group were significantly higher (*p*< 0.05) than in the healthy control group. The cartilage thickness of the femoral condyle medial, femur trochlea, femur condyle lateral central, tibia plateau medial anterior and patella facet medial inferior in the meniscus tear group were slightly higher than in the control group (*p*< 0.05). In the femur trochlea medial, patella facet medial inferior, tibia plateau lateral posterior and tibia plateau lateral central, there were significant differences in relative cartilage volume percentage between the meniscus tear group and the healthy control group (*p*< 0.05). Nineteen patients had no cartilage abnormalities (Grade 0) in the meniscus tear group, as confirmed by arthroscopic surgery, and their T2 values in most subregions were significantly higher (*p*< 0.05) than those of the healthy control group.

**Conclusion:**

The difference in articular cartilage indexes between patients with meniscus tears and healthy people without such tears can be detected by using quantitative MRI. Quantitative T2 values enable early and sensitive detection of early cartilage lesions.

## Introduction

Articular cartilage plays an important role in reducing friction, balancing load and damping in joints. However, as hyaline cartilage, articular cartilage lacks the nutritional support of blood vessels, nerves and other tissues. It can only obtain nutrition through the synovial fluid secreted by the synovium, and therefore lacks intrinsic regenerative capabilities (1–3) . As an important part of the knee joint, the meniscus also plays an essential role in maintaining the stability of the joint. Meniscal tears will cause changes in the weight-bearing capacity of the knee joint, often secondary to the destruction of the integrity of the articular cartilage and subchondral bone disease, and eventually lead to knee osteoarthritis, joint dysfunction and even disability (1, 4). Therefore, early detection of articular cartilage lesions, and intervention, is especially critical for patients with meniscus tear. Arthroscopy is an important tool for detecting articular cartilage injury, but it is limited by its invasiveness and insensitivity to early cartilage lesions without morphological changes.

Magnetic resonance imaging (MRI) is the first modality of choice for noninvasive detection of articular cartilage lesions. 3D-DESS, 3D-FLASH and 3D-SPACE sequences can display articular cartilage with high resolution and obtain accurate morphological parameters. In recent years the functional sequences have been used extensively in cartilage and cartilage repair research, such as delayed gadolinium-enhanced MRI of cartilage (dGEMRIC), T2 Mapping, T1rho and Na-MRI; they can detect biochemical and microstructural changes in the cartilage extracellular matrix even before gross morphologic changes occur (5–7). These quantitative MRI techniques make a more sensitive analysis of articular cartilage by measuring morphological and biochemical changes quantitatively, and have high accuracy and excellent repeatability (8, 9). However, as yet there has been no detailed investigation of articular cartilage morphology changes in patients with meniscus tear. The purpose of this study is to use MRI to measure the volume, thickness and T2 value of articular cartilage in patients with meniscus tear, and compare them with normal articular cartilage.

## Materials and methods

### Subjects

From June 2021 to February 2022, 32 patients with meniscus tears (17 females, 15 males; mean age: 40.16 ± 11.85 years) were selected, including 16 patients with left knee involvement and 16 patients with right knee involvement. Clinically, all patients had different degrees of knee pain, and all of them underwent preoperative MRI examination. A healthy control group of 24 knees (12 females; 12 males; mean age: 36 ± 9.14 years) was used for comparison. All subjects refrained from participation in any strenuous exercise in the 2 hours before the MRI. This was a retrospective study approved by the Medical Ethics Committee of Southwest Hospital, and we obtained the written informed consent of all subjects.

The patients in the meniscus tear group were all examined and treated under arthroscopy. The surgeons carefully examined the cartilage in the patellofemoral compartment, medial compartment, and lateral compartment, using the Outerbridge classification (10) for grading chondral lesions: Grade 0, normal articular cartilage; Grade I, chondromalacia edema or surface bubbles; Grade II, fragmentation and fissuring of articular cartilage affecting an area > 0.5 inches; Grade III, fragmentation and fissuring of articular cartilage affecting an area > 0.5 inches and Grade IV, full-thickness cartilage defect, stripped, subchondral bone exposed.

### MR examinations

All the subjects underwent MR examinations of the knee joint on a 3 T MR scanner MAGNETOM Spectra (Siemens Shenzhen Magnetic Resonance Ltd, Shenzhen, China). An eighteen-channel knee coil was used for all the MR knee scans with subjects in a supine position and feet first mode. The following MR sequences were performed: sagittal and coronal TSE T1WI (matrix 320 × 240, repetition time (TR) ms/echo time (TE) ms: 500/12, field of view (FOV) = 130*130 mm2, slice thickness = 3 mm, flip angle = 150°, total scan time = 96s); sagittal PDWI (matrix 384 × 288, TR ms/TE ms: 3000/12, FOV = 130*130 mm2, slice thickness = 3 mm, flip angle = 150°, total scan time = 131s); sagittal 3D dual echo steady state (DESS; matrix 256 × 238, TR ms/TE ms: 14.8/5.3, FOV = 150mm, slice thickness = 0.6 mm, flip angle = 25°, total scan time = 367s); T2 mapping (matrix: 384 x 288, TR/TE: 1925 ms/14 ms, FOV = 160 mm, slice thickness = 3 mm, flip angle = 180°, total scan time = 369s).

### Image processing and analysis

**Figure 1:**
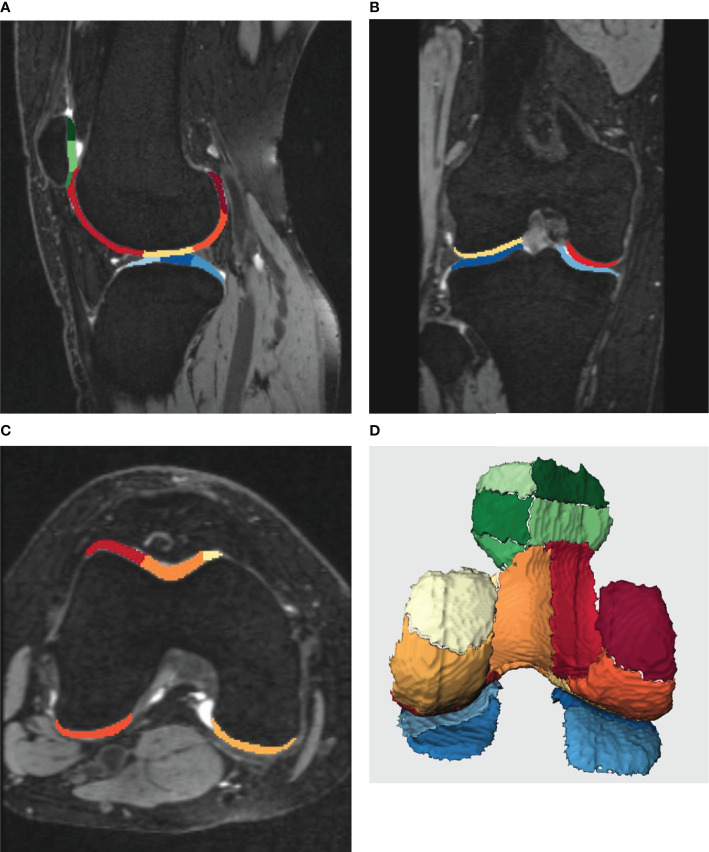
**(A-D)** shows the post-processing results of knee cartilage segmentation by Siemens KneeCaP software. Different colors represent different subregions of cartilage. **(A-C)**, the image of cartilage segmentation with bone. **(A)**, the sagittal image of the lateral tibiofemoral joint, **(B)**, the coronal image of the tibiofemoral joint, **(C)**, the cross-sectional image of the femur. **(D)**, 3D image of cartilage segmentation after bone removal (posterior anterior position).

A post-processing prototype software, KneeCaP (version 2.1, Siemens Healthcare, Erlangen, Germany), whose segmentation algorithm is based on the process and algorithm proposed by Fripp (11), was used to perform automated cartilage segmentation and measurement. The KneeCaP software segmented the sagittal MR image of the 3D-DESS sequence, divided the cartilage into 21 subregions according to the International Cartilage Repair Society (ICRS; 12; see **Table 1** and **Figure 1**), and obtained the volume and thickness results of each subregion. Then the 3D-DESS image was registered to the T2 mapping image to extract the T2 value (13). When the automatic segmentation was completed, we observed whether the segmentation result was accurate. If the result was accurate, the segmentation was considered acceptable. If not, further manual correction was needed. The relative percentage of cartilage volume was calculated as follows:


$$\text{The relative percentage of cartilage volume}(\text{RCV})=\;\left(\frac{\text{regional subregion of cartilage volume value}}{\text{total articular cartilage volume value}}\right)\times \;100\%.$$

**Table 1:** 21 subregions of knee articular cartilage automatically segmented by KneeCaP software.

Femoral cartilage	Patellar cartilage	Tibial cartilage
Femur condyle medial posterior(MP)	Patella facet lateral inferior(LI)	Tibia plateau lateral posterior(LP)
Femur condyle medial central(MC)	Patella facet lateral central(LC)	Tibia plateau lateral central(LC)
Femur condyle medial anterior(MA)	Patella facet lateral superior(LS)	Tibia plateau lateral anterior(LA)
Femur trochlea medial(TM)	Patella facet medial inferior(MI)	Tibia plateau medial posterior(MP)
Femur trochlea central(TC)	Patella facet medial central(MC)	Tibia plateau medial central(MC)
Femur trochlea lateral(TL)	Patella facet medial superior(MS)	Tibia plateau medial anterior(MA)
Femur condyle lateral posterior(LP)		
Femur condyle lateral central(LC)		
Femur condyle lateral anterior(LA)		

### Statistical analysis

SPSS 21.0 software (SPSS, version *21.0*, Chicago, IL, United States) was used for statistical analysis. Mann-Whitney-U tests were used to compare 1) the differences in relative cartilage volume percentage, cartilage thickness and T2 value between the meniscus tear group and the healthy control group; 2) the differences in relative cartilage volume percentage, cartilage thickness and T2 value between the Grade 0 subgroup in the meniscal tear group and the healthy control group. The test level α = 0.05 (*p*< 0.05) was considered statistically significant.

## Results

Arthroscopic results showed that in the meniscus tear group, there were 17 cases of medial meniscus tear (53.1%), 14 cases of lateral meniscus tear (43.8%), and 1 case of medial and lateral meniscus injury (3.1%). Cartilage lesions were evaluated according to arthroscopic Outerbridge Grade, including 19 cases of Grade 0 (59.4%), 7 cases of Grade 1 (21.9%), 3 cases of Grade 2 (9.4%), 3 cases of Grade 3 (9.4%) and 0 cases of Grade 4. The distribution of cartilage injury sites was as follows: 8 cases of medial compartment, 4 cases of lateral compartment, and 1 case of patellofemoral joint.

**Table 2** shows the median T2 value of 21 subregions of knee cartilage in the meniscus tear and healthy control groups. The T2 values of 9 subregions of the femur and 6 subregions of the tibia in the meniscus tear group were higher than those in the control group (p< 0.05). Compared with the healthy control group, the relative articular cartilage volume percentage in the meniscus tear group was statistically significant only in the following subregions: femur (TM), tibia (LP, LC) and patella (MI; p< 0.05; **Table 3**), in which the relative articular cartilage volume percentage in the patella (LP, LC) decreased slightly compared with the healthy group, and that in the femur (TM) and patella (MI) increased slightly. Compared with the healthy control group, the thickness of cartilage in the meniscus tear group increased in the following subregions: femur (MP, MC, MA, TM, TC, TL, LC), tibia (MA) and patella (MI; p< 0.05; **Table 4**).

**Table 2:** Comparison of T2 values of the articular cartilage 21 subregions between the meniscus tear group and the healthy control group.

		T2 value* (ms)		*p* value
		MT	HC	
Femur	MP	66.16	44.18	0.005
	MC	110.37	65.35	0.004
	MA	117.10	73.60	<0.001
	TM	119.83	85.76	0.002
	TC	121.83	98.51	0.003
	TL	111.26	98.03	0.023
	LP	105.55	45.05	<0.001
	LC	107.81	76.21	<0.001
	LA	127.17	92.37	0.003
Patella	LI	102.89	87.80	0.057
	LC	120.24	104.48	0.070
	LS	123.27	102.00	0.057
	MI	98.35	81.20	0.337
	MC	85.39	98.13	0.208
	MS	116.18	128.68	0.804
Tibia	LP	73.91	38.10	0.001
	LC	80.12	47.78	0.020
	LA	95.62	57.92	0.028
	MP	85.50	52.93	0.011
	MC	114.27	87.39	0.003
	MA	105.90	60.61	<0.001

*Data = median.

MT = meniscus tear group; HC = healthy control group.

p< 0.05 indicated a significant difference.

**Table 3:** Comparison of the relative cartilage volume percentage of the articular cartilage 21 subregions between the healthy control group and the meniscus tear group (only showing the subregions with significant differences).

		RCV* (%)		*p* value
		MT	HC	
Femur	TM	5.92	5.16	<0.001
Patella	MI	2.54	2.22	0.005
Tibia	LP	4.19	4.94	0.001
	LC	5.14	6.06	<0.001

RCV = the relative cartilage volume percentage.

*Data = median.

MT = meniscus tear group; HC = healthy control group.

p< 0.05 indicated a significant difference.

**Table 4:** Comparison of thickness of the articular cartilage 21 subregion between the healthy control group and the meniscus tear group.

		Thickness* (mm)		*p* value
		MT	HC	
Femur	MP	1.46	1.37	0.020
	MC	1.57	1.35	<0.001
	MA	1.71	1.59	0.021
	TM	1.74	1.55	0.001
	TC	2.23	2.01	0.009
	TL	1.92	1.68	0.006
	LP	1.57	1.48	0.089
	LC	1.75	1.55	0.003
	LA	1.56	1.46	0.074
Patella	LI	1.78	1.68	0.098
	LC	2.56	2.50	0.394
	LS	1.71	1.66	0.193
	MI	1.87	1.71	0.016
	MC	3.21	3.06	0.154
	MS	2.12	1.94	0.057
Tibia	LP	1.73	1.78	0.260
	LC	2.38	2.45	0.099
	LA	1.74	1.71	0.722
	MP	1.40	1.39	0.613
	MC	1.72	1.75	0.785
	MA	1.53	1.41	0.020

*Data = median.

MT = meniscus tear group; HC = healthy control group.

p< 0.05 indicated a significant difference.

**Table 5** shows that in the meniscus tear group, 19 patients had no cartilage abnormalities (Grade 0) confirmed by arthroscopic surgery, but the T2 values of Grade 0 cartilage in most subregions were higher than that in the healthy control group, including 9 subregions of the femur, tibia (LP, MP, MC, MA) and patella (LI, LS; *p*< 0.05). There was only a small difference in the relative cartilage volume percentage between the two groups. The relative cartilage volume percentage of the femur (TM) and patella (MI) in the Grade 0 subgroup were slightly higher than that in the healthy control group, and that of the tibia (LP, LC) was lower than that in the healthy control group (*p*< 0.05; **Table 6**). The cartilage thicknesses of the femur (MC, MA, TM, TC, TL, LC), patella (MS) and tibia (MA) in the Grade 0 subgroup were higher than those in the healthy control group (*p*< 0.05; **Table 7**).

**Table 5:** Comparison of T2 values of the articular cartilage 21 subregion between the meniscus tear group Grade 0 subgroup and the healthy control group.

		T2 value* (ms)		*p* value
		MT(G0)	HC	
Femur	MP	73.53	44.18	0.012
	MC	118.80	65.35	0.019
	MA	125.69	73.60	0.003
	TM	131.88	85.76	0.002
	TC	129.95	98.51	0.002
	TL	117.62	98.03	0.026
	LP	105.46	45.05	<0.001
	LC	115.38	76.21	<0.001
	LA	125.51	92.37	0.014
Patella	LI	102.91	87.80	0.048
	LC	122.39	104.48	0.115
	LS	124.85	102.00	0.035
	MI	100.25	81.20	0.163
	MC	91.32	98.13	0.903
	MS	121.72	128.68	0.883
Tibia	LP	79.20	38.10	0.002
	LC	75.12	47.78	0.067
	LA	94.67	57.92	0.067
	MP	102.33	52.93	0.001
	MC	118.10	87.39	0.006
	MA	119.83	60.61	0.001

*Data = median.

MT(G0) = meniscus tear group Grade 0 subregion; HC = healthy control group.

p< 0.05 indicated a significant difference.

**Table 6:** Comparison of the relative cartilage volume percentage of the articular cartilage 21 subregion between the healthy control group and the meniscus tear group Grade 0 subgroup (only showing the subregions with significant differences).

		RCV* (%)		*p* value
		MT(G0)	HC	
Femur	TM	5.91	5.16	<0.001
Patella	MI	2.62	2.22	0.005
Tibia	LP	4.10	4.94	0.002
	LC	5.35	6.06	<0.001

RCV = the relative cartilage volume percentage.

*Data = median.

MT(G0) = meniscus tear group Grade 0 subgroup; HC = healthy control group.

p< 0.05 indicated a significant difference.

**Table 7:** Comparison of thickness of the articular cartilage 21 subregion between the healthy control group and the meniscus tear group Grade 0 subgroup (only showing the subregions with significant differences).

		Thickness* (mm)		*p* value
		MT(G0)	HC	
Femur	MC	1.52	1.35	<0.001
	MA	1.71	1.59	0.017
	TM	1.68	1.55	0.005
	TC	2.20	2.01	0.023
	TL	1.96	1.68	0.013
	LC	1.67	1.55	0.010
Patella	MS	1.73	1.94	0.019
Tibia	MA	2.38	1.41	0.041

*Data = median.

MT(G0) = meniscus tear group Grade 0 subgroup; HC = healthy control group.

p< 0.05 indicated a significant difference.

## Discussion

In our study, arthroscopy showed that most of the articular cartilage in the meniscus tear group was normal (59.4%), and 13 (40.6%) patients in the meniscus tear group had grade I-III cartilage lesions. The cartilage injuries accounted for 40.6%, of which the primary injury was 21.9%, and the injury site was mostly located in the medial compartment of the knee joint (61.5%). This finding is consistent with previous studies that cartilage volume is reduced in the meniscal tear group compared to those without an absence of tears, especially in the medial compartment of the knee, as suggested by Berthiaume et al. (14). The T2 values of all subregions of the femur and tibia in the meniscus tear group were higher than those in the healthy control group, which could reflect the relationship between meniscus tear and cartilage lesion. The meniscus and articular cartilage of the knee joint are highly correlated in embryology, anatomy and function, which explains why the pathological changes in one also affect the other. The disorder of meniscus structure will influence the distribution of strength, and the pressure load on articular cartilage will increase, resulting in cartilage lesion (15, 16). This close relationship is also confirmed by the effect of meniscus lesion on articular cartilage (14, 17, 18). The individual differences in cartilage volume of the knee joint were affected by many factors, the volume being positively correlated with body weight, height, leg length, and foot size (19, 20). In this study, the relative cartilage volume percentage in each subregion of the knee was used to detect the differences in cartilage volume between patients with meniscus tear and healthy volunteers, which can reduce the somatotype difference in cartilage volume to a certain extent. The results showed that the relative cartilage percentage in the femur (TM) and patella (MI) subregions in the meniscus tear group were higher than that in the healthy control group, while in the tibia (LP, LC) subregion it decreased. This is inconsistent with Berthiaume’s findings (14). We speculate that the reasons could be: 1) The change in cartilage volume depends on the grade of cartilage lesion. Grade I cartilage lesion shows cartilage swelling, resulting in cartilage volume slightly increasing, while grade II-IV cartilage injury shows different degrees of cartilage defect, resulting in cartilage volume reduction. In our meniscus tear group, normal cartilage and grade I cartilage lesion accounted for the largest proportion; 2) The relative cartilage volume percentage could only reduce the influence of somatotype difference to a certain extent (21), but it could not completely eliminate other influencing factors. In the horizontal research, when the cartilage injury is mild, the relative cartilage volume percentage may not accurately reflect the change in cartilage volume. All these hypotheses need to be confirmed by further research; 3) Automatic segmentation with the prototype KneeCaP software on severely injured articular cartilage was not accurate enough. The automatic segmentation software mistakenly regarded the synovial tissue around the cartilage as cartilage tissue, resulting in a larger volume of cartilage segmentation than its actual volume (21). Our study showed that the thickness of cartilage in patients with meniscus tear was higher than that in healthy controls in most subregions of the femur and some subregions of the patella and tibia, and the difference was statistically significant. This result indirectly reflected the swelling of cartilage in patients with meniscus tear.

By comparing the differences in T2 value, cartilage volume percentage and cartilage thickness between patients with meniscus tear Grade 0 and the control group, we found that the T2 value of most subregions of cartilage in the former was higher than in the latter, which suggests that there were biochemical changes in articular cartilage at an earlier stage than morphological changes in patients with meniscus tear. However, it is difficult to observe this subtle change using arthroscopy. One advantage of cartilage MRI examination is that the T2 value of cartilage can indirectly reflect the changes in biochemical components. The value of T2 is mainly affected by the water content and collagen fibers. The increase in T2 value usually represents an increase in water content and a loss of collagen anisotropy (22, 23). Previous studies have confirmed that articular cartilage lesion will be accompanied by an increase in T2 value (24). In this study, we found that the T2 values of articular cartilage in patients with meniscus tears increased in most subregions, which also indirectly verifies the above conclusions.

In contrast to some previous studies, where the region of interest was sketched manually, we performed automatic segmentation in all subjects, which has excellent reproducibility and is not affected by inter-observer variation (21). It also leads to a reduction in the time and effort involved. However, the T2 value of articular cartilage we obtained is generally higher than in previous studies, mainly because of our different methods of measuring T2 value and registration algorithm (25, 26).There are several limitations to our study: 1) The sample size was small and the degree of cartilage lesion in the meniscus tear group was uneven, especially the large proportion of grade 0, which is why we did not further discuss the classification of cartilage lesion in the groups. Therefore, it is necessary to further expand the sample size in future research to improve its credibility. 2) In our study, various indexes of articular cartilage in the two groups were compared horizontally, which made it difficult to avoid the errors in T2 value caused by individual differences, such as age and BMI (27, 28). In the follow-up, we will longitudinally follow up the changes in cartilage in patients with meniscus tear after arthroscopic treatment.

To sum up, there are differences in various indexes of articular cartilage between patients with meniscus tear and healthy subjects, which can be detected by MRI quantitative techniques. Quantitative magnetic resonance T2 technology has a higher sensitivity to early cartilage lesion than arthroscopy. Morphological quantitative parameters such as cartilage volume and thickness are relatively less sensitive to mild cartilage lesion, and morphological quantitative parameters of cartilage are more suitable for longitudinal research.

## Data availability statement

The original contributions presented in the study are included in the article/supplementary material. Further inquiries can be directed to the corresponding authors.

## Ethics statement

The studies involving human participants were reviewed and approved by The Medical Ethics Committee of Southwest Hospital. The patients/participants provided their written informed consent to participate in this study. Written informed consent was obtained from the individual(s) for the publication of any potentially identifiable images or data included in this article.

## Author contributions

EC, WH, HW and WC finished this article. EC, WH, JL and YL acquired the data together. EC, WH, QY and WC designed this study. All authors researched the literature and analyzed the results together.

## Acknowledgments

The authors would like to thank Axel Newe from Methodpark, Erlangen, Germany for his work on the KneeCaP prototype and International Science Editing for editing this manuscript.

## Conflict of interest

The authors declare that the research was conducted in the absence of any commercial or financial relationships that could be construed as a potential conflict of interest.

## Publisher’s note

All claims expressed in this article are solely those of the authors and do not necessarily represent those of their affiliated organizations, or those of the publisher, the editors and the reviewers. Any product that may be evaluated in this article, or claim that may be made by its manufacturer, is not guaranteed or endorsed by the publisher.
